# Consumer Perceptions of the German Food‐Based Dietary Guidelines and the Complementary Potential of AI‐Based Nutrition Tools

**DOI:** 10.1002/fsn3.72242

**Published:** 2026-08-10

**Authors:** Nicolas Hartmann, Philipp Sprengholz

**Affiliations:** ^1^ Institute of Psychology University of Bamberg Bamberg Germany; ^2^ Institute for Planetary Health Behavior University of Erfurt Erfurt Germany; ^3^ Health Communication Working Group Bernhard Nocht Institute for Tropical Medicine Hamburg Germany

**Keywords:** artificial intelligence, concept evaluation, food‐based dietary guidelines, nutrition communication, qualitative research

## Abstract

Food‐based dietary guidelines (FBDGs) are a key instrument for communicating healthy nutrition principles that are implemented in most countries. However, adherence remains low, and recent literature highlights the need for improvements. This exploratory study investigated consumer perceptions of the German FBDGs, focusing on strengths and areas for improvement. Ten individuals with diverse demographics, nutrition knowledge, and dietary motivation participated in semi‐structured interviews. Participants evaluated the content and design of the FBDGs regarding both comprehensibility and applicability. Additionally, participants provided feedback on a proposed mock‐up illustrating the concept of an AI‐based feedback tool aimed at enhancing individualization and contextual aspects. Overall, participants expressed more criticism than approval. Although the FBDGs were generally considered easy to understand, many found them difficult to translate into daily practice. Participants perceived the FBDGs as (a) too general for individualized diets and (b) too theoretical, offering little support for behavior change. Positive feedback mainly referred to the simple and understandable language. The mock‐up was received more positively, though some criticism regarding individualization was raised. The findings suggest that while the German FBDGs effectively illustrate healthy eating principles conceptually, they fall short in supporting real‐life implementation. The presented AI‐based tool represents a promising step toward more practical and individualized guidance but may still not be sufficient to induce substantial changes to behaviors. Future improvements to national dietary guidance may therefore require the development of complementary, behaviorally oriented programs that go beyond informational approaches and address the practical realities of everyday nutrition.

## Graphical abstract

This exploratory qualitative study examined consumer perceptions of the German Food‐Based Dietary Guidelines (FBDGs) using semi‐structured interviews with 10 participants. Although the FBDGs were broadly comprehensible, implementation proved difficult for many, as guidelines were perceived as too general and theoretical. A conceptual AI‐based feedback tool mock‐up was presented and positively received, representing a promising yet empirically untested complementary approach.

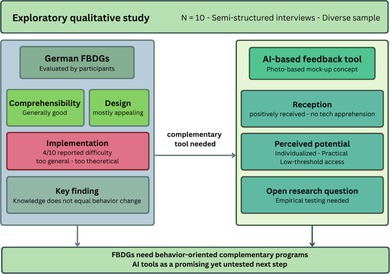

## 1 Introduction

Food‐based dietary guidelines (FBDGs) have come to be one of the most used resources for information about healthy nutrition at a national level. They have been recommended for many years by global health organizations, such as the World Health Organization (WHO) and the Food and Agriculture Organization of the United Nations (FAO) (FAO and WHO 1992). According to the FAO, more than 100 countries have implemented their own FBDGs (FAO 2025).

FBDGs are commonly evidence based and aim to translate quantitative nutritional goals and reference values into qualitative recommendations that are easy to understand and comparable with one's own diet. They are intended to be a low threshold way to promote nutritional knowledge and support healthier eating habits (FAO and CFNI 2007; Albert et al. 2007; Schneeman 2003). For example, the German FBDGs, developed by the German Association for Nutrition (Deutsche Gesellschaft für Ernährung, DGE) and updated in 2024 (DGE 2024), consist of (a) 11 written recommendations (DGE 2025a), (b) the *nutrition circle* as a visual representation of a balanced diet, and (c) the *orientation table*, showing exact reference quantities based on an assumed daily energy intake of 2000 kcal (DGE 2025b).

New literature and updated guideline models have also begun to address environmental aspects in FBDGs (Dooren et al. 2024; Herforth et al. 2019; Mazac et al. 2021; Springmann et al. 2020). This change reflects a growing recognition of nutrition‐related emissions, land, and water use, all of which contribute to climate change and biodiversity loss, endangering both the environment and human health (Crippa et al. 2021; Stoll‐Kleemann and Schmidt 2016). The updated German FBDGs (DGE 2024) represent one of the first practical applications incorporating such aspects. A mathematical optimization model was used to incorporate health and environmental aspects, while not deviating too much from Germany's current diet, based on the latest German National Nutrition Survey (Nationale Verzehrsstudie II; NVS II). Full adherence to these recommendations has been estimated to yield health gains of 2.67–4.37 disability‐adjusted life years per person, as well putting Germany on track to achieve the Intergovernmental Panel on Climate Change target of a 45% reduction in diet‐related greenhouse gas emissions by 2030 (DGE 2024).

Studies have shown that, despite their scientific grounding and policy relevance, adherence to FBDGs is poor (Brown et al. 2011; Leme et al. 2021). Leme et al. (2021) found that fewer than 50% of national populations adhere to even one of their FBDG recommendations. This limited impact is often attributed to two key challenges: (a) FBDGs may not be close enough to individuals' everyday lives and dietary conditions (EFSA 2010; Bechthold et al. 2018; Montagnese et al. 2015); and (b) FBDG developers often lack concrete plans on implementation dissemination strategies (Wijesinha‐Bettoni et al. 2021).

Our study aims to exploratively examine these shortcomings through qualitative research. For this, we used semi‐structured interviews to ask 10 individuals with different demographic characteristics and lifestyle aspects about the comprehensibility and applicability of the German FBDGs. We also introduced the participants to the concept of a photo‐based, AI‐supported feedback tool that could potentially address some of the identified limitations by offering individualized, context‐sensitive dietary feedback. This approach was intended to explore the perceived potential of digital tools to enhance traditional FBDGs while remaining within the scope of an informational approach.

## 2 Methods

Participants were recruited through a convenience sampling approach. Students of the University of Bamberg were asked whether they or their family members would be willing to participate in an interview about the current German FBDGs. From approximately 30 potential participants, 10 were selected to achieve a diverse sample across age groups (young, middle‐aged, and elderly), occupational status (students, employed, and retired), and gender. Data collection was considered sufficient when repeated interviews no longer generated substantially new categories or themes relevant to the study aim. We planned to conduct an initial set of 10 interviews, with the option to continue if saturation had not been reached. As later interviews largely confirmed previously identified themes, we considered the 10 completed interviews to be sufficient. All 10 participants received financial compensation of €20, regardless of whether they completed the interview. Prior to recruitment, participants were informed about the interview procedure, including audio recording. Verbal informed consent was obtained and documented at the beginning of each recorded session. Ethical approval was obtained from the University of Bamberg's IRB (#2025‐12/109). The interviews followed a semi‐structured format and took about 30 min to complete.

At the beginning of each session, participants were asked to provide demographic and nutritional background, specifically their gender, age, occupational or student status, and dietary particularities related to allergies, intolerances, or restricted diets. Afterward, participants were asked to evaluate their nutritional knowledge. To verify if self‐reported knowledge aligned with established nutritional standards, participants were also asked which aspects of their diets they considered “rather healthy” or “rather unhealthy”. To conclude this first part of the interviews, participants were asked (a) to describe the overall quality of their diet in terms of health, (b) to reflect on whether healthy nutrition is important to them, (c) whether they would like to change anything about their current diet and, if so, (d) whether they felt capable of doing so.

In the second part of the interview, participants were instructed to work through the German FBDGs using a think‐aloud approach. They were handed printed versions of the three parts of the guidelines: (a) the written recommendations, (b) the *nutrition circle*, and (c) the *orientation table*. They were asked to read through them and verbalize their thoughts. This was followed by targeted questions about the content, design, and applicability of the German FBDGs. Content questions included whether participants had understood the recommendations, which parts they considered rather easy or rather difficult to comprehend, and what could be done to facilitate better understanding. Design questions included what participants perceived to be well designed, what they considered poorly designed, and what could be changed to improve overall design. Applicability questions included whether participants knew how to implement the recommendations in their daily lives and what changes or additions could support them in transferring the guidelines more easily into everyday practice. When participants stated dietary particularities (e.g., allergies) at the beginning of the interview, they were asked if these were well addressed in the German FBDGs.

**FIGURE 1:**
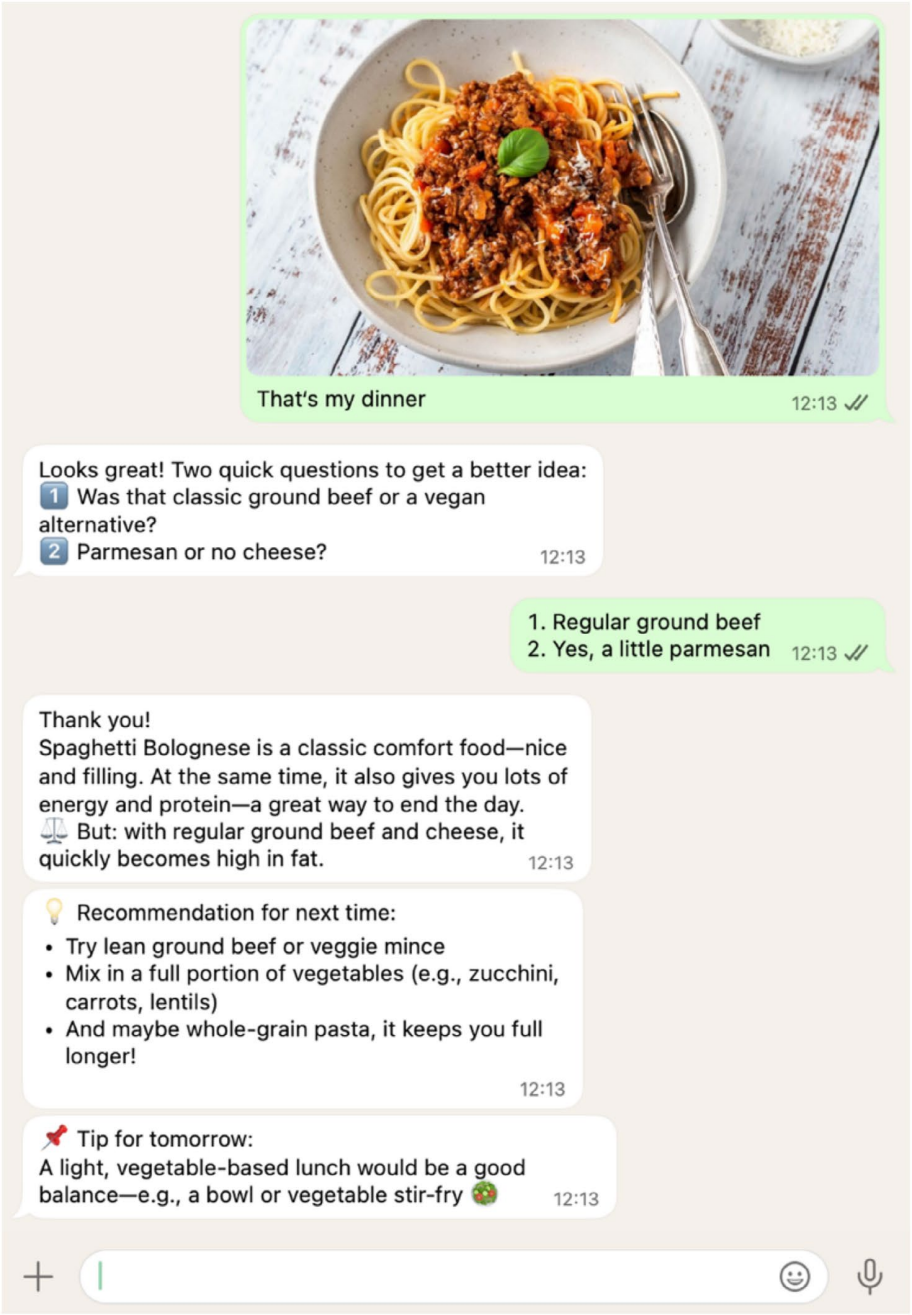
Prototype of the photo‐based feedback tool. English translation. Original material was presented in German language.

In the third part of the interview, participants were introduced to the concept of a photo‐based feedback tool. Calorie‐tracking applications have recently begun to incorporate AI‐supported photo analysis of meals, shifting the focus away from collecting and analyzing caloric and nutrient values toward providing practical, language‐based feedback accessible to individuals with varying levels of nutritional knowledge. To illustrate the concept, a printed example of a WhatsApp chat was presented, showing how a user might send a photo of a meal and receive a response providing feedback (Figure 1). The proposed interaction sequence included the following steps: (a) the user sends a photo of their meal, (b) the application requests additional information if needed for clearer analysis and then (c) provides a health‐related assessment of the meal, (d) suggests possible alterations that could be made to the recipe to support healthy eating habits, and (e) recommends a complementary recipe for the next meal. Participants were asked to react to the example using the think‐aloud approach. Afterward, they were asked how they liked the concept and whether they would consider using such a tool themselves. They were also asked what kind of feedback they would find most helpful and which aspects might prevent them from using such a tool on a regular basis.

Reporting of this study followed the Standards for Reporting Qualitative Research (SRQR) guidelines (O'Brien et al. 2014). Data analysis followed a qualitative content analysis approach combining deductive and inductive elements. Categories were derived deductively from the interview guide, while additional codes were developed inductively to capture emerging ideas and themes. To ensure coding quality, the deductive category framework was developed collaboratively by both authors, while inductive codes were developed by the first author and reviewed by the second. The researchers were aware that their academic background and interest in nutrition communication and digital health tools could influence data interpretation. To mitigate this potential bias, the interviewer deliberately refrained from expressing their own perspectives or opinions during data collection. Furthermore, emerging codes and themes were discussed between both authors, and interpretations were checked against the original interview transcripts to ensure they remained grounded in participants' accounts. Interviews were conducted and analyzed in German. Quotes were translated into English by the first author, who took particular care to preserve the original meaning without introducing translation‐related changes to the content. The original German quotes are available in the online supplement.

## 3 Results

### 3.1 Participant Characteristics

A total of 10 individuals participated in the interviews. The sample included four male participants (aged 22, 22, 26, and 54) and six female participants (aged 23, 25, 39, 53, 56, and 76). Four participants (two male, two female) were university students, five (two male, three female) were employed, and one female participant was retired. The following paragraphs detail relevant demographic characteristics and nutrition‐related behaviors of each participant in the order in which the interviews were conducted.

Participant 1 (P1) was a 22‐year‐old male student with a strong interest in sports, who had fostered a high level of nutritional awareness. He prioritized sufficient energy and nutrient intake to support his football training and primarily aimed to avoid weight loss. Recently, he managed to overcome a tendency to snack in the evening. He considered himself self‐efficacious in his ability to adapt his dietary behavior when necessary.

Participant 2 (P2) was a 22‐year‐old male student who reported substantial nutritional knowledge and integrated it consistently into his daily life. He regularly practiced meal‐prepping and tended to prioritize nutrient density over taste. Looking ahead, he perceived himself as capable of adopting new dietary practices if they were to become standard recommendations for health.

Participant 3 (P3) was a 23‐year‐old female student who follows a vegan diet. She possessed a foundational understanding of nutrition but reported challenges in translating this knowledge into sustained, health‐promoting habits. She expressed a desire to improve in this area but did not see herself as self‐efficacious in doing so.

Participant 4 (P4) was a 54‐year‐old male engineer. He demonstrated a solid understanding of nutrition and generally maintained a balanced diet. However, he occasionally prioritized taste over health, which led to occasional snacking, consumption of unhealthy meals, and alcohol intake. He believed he would be able to improve these habits if necessary.

Participant 5 (P5) was a 53‐year‐old female who worked in an office. She reported limited nutritional knowledge and generally maintained rather unhealthy eating habits, such as consuming sweetened drinks and snacks. Although she did not wish to change her dietary behavior, she believed she could make changes if they were necessary for health reasons.

Participant 6 (P6) was a 39‐year‐old female who worked as a secretary. Due to a histamine intolerance, she had educated herself extensively on nutrition and followed a highly health‐conscious diet. Given the restrictions associated with her condition, she saw little reason or possibility to further modify her current eating behavior.

Participant 7 (P7) was a 25‐year‐old female student who reported good nutritional knowledge and generally applied this in her daily life. However, she noted that her reliance on her university's canteen, which did not always offer healthy options, sometimes limited her ability to maintain her preferred eating habits. She believed that in the future, she would be able to rely less on the canteen and cook more often.

Participant 8 (P8) was a 26‐year‐old male working in chemistry research. He reported a good level of nutritional knowledge and a moderate level of implementation. While he valued a healthy diet, he struggled with consistently eating a sufficient amount of fruits and vegetables and cooking for himself regularly, primarily due to time constraints associated with his work.

Participant 9 (P9) was a 76‐year‐old retired female who reported a high level of nutritional knowledge and adhered to a healthy diet. Her only indulgences were a small sweet snack and a beer in the evenings. She was satisfied with her current dietary routine and did not foresee making changes, nor did she consider herself likely to adopt new behaviors.

Participant 10 (P10) was a 56‐year‐old female employed in an office. Recently, she had increased her nutritional awareness and decided to place greater emphasis on health and well‐being. As a result, she had made several positive changes to her diet, such as having a more nutrient‐dense breakfast and eating fewer processed foods and more plant‐based foods. She felt confident in her ability to implement further adjustments if necessary.

### 3.2 Evaluation of the German FBDGs

#### 3.2.1 Comprehensibility

Regarding comprehensibility, all 10 participants reported a good to very good level of understanding of the FBDGs' content. When differentiating between the three content components, the recommendations and the *nutrition circle* were generally well received. Several participants highlighted the use of simple language (P1) and the illustrative character of the *nutrition circle* (P4). Despite all the positive feedback, P3 considered the *nutrition circle* to be uninformative, stating, “I find it weird. It's especially a shame they didn't include percentage quantities.”

Conversely, the *orientation table* was perceived to be rather challenging. P3 and P4 reported a moderate to low understanding of the *orientation table*, with P3 attributing this to a lack of nutritional background and P4 to the table's detail, which did not invite engagement. Across participants, the reference values were often described as difficult to comprehend (P1, P3, P4, P7, P8). As P7 explained, “It just sounds like so much […] You lose track, because when this is laid out in front of you, it gives you the feeling that you should be eating something all the time.” The practical relevance of the *orientation table* was also brought into question, since the assumed average energy intake of 2000 kcal/day was regarded by many participants to be unrepresentative of their diet (P1, P2, P3, P8). P2 suggested that the FBDGs should better emphasize that the reference quantities are not rigid targets. Moreover, he expressed the desire for more educational content, especially related to macronutrients and their distribution in foods.

Concerns about inclusivity were also raised. Several participants felt that the FBDGs insufficiently addressed individuals with specific dietary needs, such as intolerances and special diets like veganism (P3, P6, P9). P6 summarized this sentiment, noting, “That's only true for someone who has no problems at all and who really eats everything. […] And I think there are a lot of people who simply prefer certain things and would perhaps say, ‘Give me a few tips on how I can still incorporate that somehow’.”

Beyond comprehensibility, participants questioned the correctness and consistency of certain recommendations. Some participants criticized omissions, like the lack of reference to heavy metal contamination in some fish species (P2, P7, P8) or not addressing alcohol at all (P4). On the other hand, P3 and P8 questioned the need for juice in the *orientation table*. Other participants highlighted contradictions: P7 noted that the *orientation table* recommends equal amounts of spreadable fats and vegetable oils, even though the recommendations advise favoring vegetable oils. P1 pointed out the tension between recommending a predominantly plant‐based diet and suggesting daily consumption of dairy products, noting that the dairy products could be supplemented more easily than implied by the FBDGs. Other recommendations were perceived as unrealistic or unclear, such as the definition of “portions” in the fruit and vegetable guideline (P2, P3, P7, P8), the intake of only one egg per week (P2, P7), or the recommended daily water intake of 1.5 L, which was considered too low (P2, P8).

Participants also expressed critical views regarding the scope and framing of the FBDGs. These statements included the perception that animal‐based foods are underrepresented in the *nutrition circle*, at least for athletes (P2), that there should be distinctions between red and white meat (P2, P8), between fruit and vegetables (P8), and between different vegetables (P3). Finally, participants proposed ways to enhance the FBDGs' comprehensibility and relevance. These included providing clearer explanations for why specific foods and nutrients are important and how processing impacts nutrient quality (P3). P4 emphasized that subjective well‐being may be more meaningful than adherence to specific numerical targets.

In summary, nearly all participants reported that the recommendations and the *nutrition circle* were both comprehensible and appealing, while the *orientation table* was generally perceived as difficult to understand. In particular, quantitative information was viewed as either unclear or overly detailed and theoretical. Overall, participants perceived the FBDGs as providing insufficient or rather irrelevant information and as using impractical language. They expressed a desire for greater individualization, inclusivity and practical relevance, accompanied by terminology and measurements that better reflect daily dietary behavior.

#### 3.2.2 Design

Overall, most participants considered the German FBDGs' design to be attractive. P3 described the design as visually pleasing but somewhat empty in content, explaining, “To me it gives the impression that the images are rather there to lighten the mood and make it more appealing to look at, than to actually convey a message. I think they could be used in a much more meaningful way to make it easier to understand.” P2 particularly emphasized that both the recommendations and the *nutrition circle* were well presented and that the *nutrition circle* effectively illustrates that there are different ways to achieve a healthy diet.

However, not all participants favored the current visualization. P6 preferred the earlier pyramid version they had learned in school over the current *nutrition circle*, perceiving the pyramid format as more informative. The *orientation table* was evaluated by multiple participants as being poorly designed (P2, P6, P10).

Several participants proposed ideas to improve visual presentation and user engagement. P10 suggested integrating the *orientation table* into the *nutrition circle* as interactive pop‐up information. P3 recommended the use of more realistic images that reflect everyday foods and highlight key features like whole grains. P1 proposed presenting the *nutrition circle* as a weekly shopping basket to improve practical applicability.

Overall, while the design of the FBDGs was viewed positively, participants emphasized that improved visualization through more practical, relatable, and interactive elements could enhance comprehension.

### 3.3 Implementation

Participants reported mixed estimations. While five participants (P2, P6, P8, P9, P10) reported their ability to follow the recommendations to a substantial degree, four participants (P1, P3, P5, P7) stated that they were unable to do so. P4 reported the ability on a knowledge level but difficulty in sticking to it on a regular basis.

Participants who reported difficulty with implementation described the FBDGs as too theoretical and detached from everyday behavior (P1, P3). P1 explained that he does not consider the guidelines during grocery shopping, and P3 described the FBDGs as “far removed from reality.”

Several participants mentioned that parts of the recommended diet were perceived as too expensive, particularly fish (P1, P3, P7), vegetable oils (P1, P3, P7), nuts (P1, P3), fruits and vegetables (P1), and whole grain products (P3). Among participants struggling with implementation, cost‐related concerns were frequently mentioned; notably, three of the four participants in this group raised financial barriers as a relevant issue. This suggests that affordability represents a salient obstacle. However, as socioeconomic status was not systematically assessed in this study, it remains unclear to what extent these concerns reflect broader differences related to socioeconomic status rather than individual perceptions of cost.

Additionally, contextual and structural barriers were mentioned. P5 indicated that she is not able to eat according to the FBDGs due to her work's environment offering no easily accessible options for a healthy lunch. P7 did not know how to integrate specific recommended foods into her daily eating routine.

In summary, implementation would be difficult for four of our 10 interviewees, primarily due to the guidelines' perceived theoretical orientation, limited practical guidance, and the financial burden of some of the recommended foods.

#### 3.3.1 Suggestions for Improving the German FBDGs

Participants provided numerous ideas for how the German FBDGs could be made more effective and applicable in everyday life. P3 and P9 emphasized the need for guidelines to better address individuals who cannot consume certain foods by including suitable alternatives. A broader call for individualized recommendations was voiced by many participants (P1, P2, P4, P6, P8). P8 proposed developing a personalization tool based on the concept of the Wahl‐O‐Mat, a well‐known German election decision aid. P4 suggested an individualized monitoring tool that alerts users to potential nutrient deficiencies.

Participants' ideas largely clustered around three themes: (a) better alignment with daily eating behavior, (b) the inclusion of information about grocery shopping, and (c) guidance on how eating habits develop and can be changed.

Regarding practical applicability, several participants wished for more concrete and practical measures instead of abstract gram specifications. P3 and P8 suggested using household measures, such as slices of cheese or ham; P3 explained, “Nobody wants to use a scale while cooking, wondering whether they use enough lentils or not. It would be much easier to say you should eat one plate of lentils per week, that would be much easier and more applicable.” P7 would have liked everyday examples of what a day following the German FBDGs might look like. P6 requested information on how and when to consume the recommended nutrients, and P7 asked for distinctions between foods suitable as snacks or as main meal components. This adds to the proposal of P1 for situational recommendations, such as seasonal meal options or cost‐effective product alternatives.

Multiple participants emphasized that the FBDGs should use examples based on products commonly available in grocery stores. P1 noted that the guidelines focus too heavily on abstract food components rather than actual consumer products and suggested addressing marketing and placement strategies that grocery shops commonly use. P10 similarly expressed interest in receiving guidance on grocery shopping and diet planning.

Beyond dietary content, several participants also suggested addressing behavioral aspects of nutrition. P4 stated that the primary reason for unhealthy eating habits is not a lack of knowledge, but the implementation. Therefore, he proposed that the FBDGs should include information about how eating habits are developed and how they can be changed. Similarly, P5 suggested providing preparation strategies for stressful situations, such as short lunch breaks.

Participants also expressed an interest in specific recipe ideas that comply with the recommendations to facilitate their application in everyday life (P2, P3).

Overall, participants emphasized that the German FBDGs could be made more effective by offering individualized, practical, and behavior‐oriented guidance, extending beyond bare nutritional knowledge to be applicable to everyday eating contexts.

### 3.4 Comments on the Photo‐Based Diet Recommendation Tool

Participants also evaluated the presented photo‐based diet recommendation tool. In general, participants felt positive about the tool. Several participants (P1, P4, P6, P7) appreciated that the tool communicates feedback in a motivational manner without harsh criticism. P2, P3, P4, P7, and P8 highlighted the tool's simplicity, accessibility, and straightforward design, which would be easy to integrate into existing routines. P3 liked the applicability in concrete situations, detailed information, and attention to plant‐based nutrition. Conversely, P8 noted that receiving criticism after every meal could be discouraging, and P2 doubted the precision of the photo analysis. P10 found the tool interaction to be too long.

Looking at specific tool components, two participants appreciated the constructive criticism of the photographed meals (P1, P10). The majority responded positively to the suggestion of healthier alternatives (P1, P2, P3, P5, P6, P10) and to the “tip for the next day” feature (P1, P2, P3, P6, P7, P10).

Regarding potential usage, P1, P2, P3, P6, and P9 stated that they would primarily use the tool as inspiration for meal planning. Additionally, P1 reported that he would use it for more precise knowledge about his diet. P4 would use the tool as monitoring device to support changes in undesired eating habits, whereas P7 indicates she would use it only if she had a specific goal, such as losing weight. P10 would not use the tool regularly, as it would require too much effort for her age.

Participants also provided several ideas for further optimization, with one major theme being customization. P7 and P8 wanted the inclusion of individualized goals, such as losing weight or gaining muscle mass, while others suggested options for dietary preferences or restrictions, including food intolerances (P6) and plant‐based diet (P3). Participants also proposed ideas for additional integration features: specific recipes (P1, P2, P3, P9), the option to track groceries purchases (P4), and precise gram quantities (P2), as well as information on macro‐ and micronutrient distributions (P4, P8). P4 further thought of adapting recommendations to individual schedules (e.g., training days) and pairing the tool with smart watches, potentially using gamified elements similar to step‐counting applications.

Concerning the feedback mechanism, P6 and P8 did not want to be criticized every time they interacted with the program, but would rather receive critical feedback occasionally. P4 suggested an adaptive system that adjusts the frequency and tone of feedback according to the user's daily willingness to adhere to the recommendations.

In summary, participants valued the tool's motivational tone, accessibility, and practical orientation but emphasized the need for more individualized, flexible, and adaptive functions. Further improvement could be made through customization, integration of personal goals and technologies, and a balanced feedback system.

## 4 Discussion

In our study, participants generally estimated the German FBDGs as easy to comprehend, aside from the *orientation table* which presented a challenge to some participants. A similar pattern was found with regard to design, seeing the recommendations and the *nutrition circle* as visually appealing and the *orientation table* as rather overwhelming. According to the collected feedback, comprehensibility could be further improved by incorporating more practical information and visualizations, as well as by expressing quantities in household measurements instead of grams. Additional improvements could be achieved by developing complementary tools that address individual differences reflecting real life, such as gender, activity level, food intolerances, non‐omnivore diets, socioeconomic factors, and work–life aspects, which go beyond the intended scope of population‐level guidelines.

The findings align with existing research indicating that FBDGs are often perceived as insufficiently tailored to individual circumstances and disconnected from everyday behavior (EFSA 2010; Bechthold et al. 2018; Montagnese et al. 2015; Wijesinha‐Bettoni et al. 2021), highlighting the need for individualized and integrative approaches that complement, rather than replace, the population‐level guidance that FBDGs are designed to provide. Such an approach may foster stronger behavioral engagement and more sustained dietary change.

An attempt to implement some of the desired adaptations can be seen in the investigated photo‐based evaluation tool. Feedback on the concept was consistently positive and substantially better than that received for the FBDGs. However, as the tool was presented only as a printed mock‐up rather than a functional application, these reactions reflect perceived potential rather than actual user experience. Reported ideas for further optimizing this idea were to have even more individualization and adaptation possibilities and not to use too much criticism. No participant, including older participants, expressed any fear or discomfort toward artificial intelligence in general or toward sharing meal photos. This suggests that an AI‐based tool similar to the presented concept would likely be broadly accepted and would not evoke high levels of technological apprehension among users. Combining this acceptance with the advancing possibilities of AI in personalized health support, future research should explore the development and empirical testing of AI‐based dietary feedback tools as widely implementable complementary approaches to national FBDGs.

Nevertheless, our proposed tool idea also has its limitations. As participants mainly reported that they would use the tool for inspiration, like the FBDGs, it may not lead to substantial changes in food‐related behaviors. Only two participants reported that they would use it for intentional change, either toward habitual change (P3) or physical goals (P7). In this study, most participants reported a rather healthy diet or self‐efficaciousness in changing their diet if desired. Therefore, this generally optimistic sample may have biased the findings, as few participants appeared to represent individuals who would substantially benefit from the presented tools. Furthermore, the convenience sample was drawn from one university network in a single German city, which constrains the transferability of findings beyond the educational and socioeconomic profile represented in this sample. It should also be noted that some participants contributed more extensively than others to certain themes, as the interviewer prioritized a natural conversational flow over balanced input per question. Differences in response richness are reflected in the varying number of participant codes cited per statement.

One major finding of this study is that there appeared to be no association between comprehension of the FBDGs, a positive evaluation of the design, or existing nutritional knowledge and the actual implementation of the guidelines in daily life. This suggests that cognitive and aesthetic aspects of dietary communication alone may not translate to behavioral adherence, highlighting the persistent gap between nutritional knowledge and practical application.

This underscores a central dilemma of public health policies: While FBDGs fulfill their intended purpose as population‐level information tools, informational approaches alone appear insufficient to elicit substantial dietary behavior changes (Brown et al. 2011; Leme et al. 2021). Given that countries such as Germany have not implemented large‐scale complementary programs addressing behavioral, environmental, and socioeconomic determinants of eating behavior, the overall impact of national nutrition promotion efforts remains constrained.

The proposed photo‐based evaluation tool may represent a step toward an informational approach more closely aligned with users' everyday behavior and capable of providing practical, context‐related feedback. Nevertheless, so far it cannot be considered a sufficiently behavior‐oriented intervention and would therefore not serve as a stand‐alone solution to the identified shortcomings of the FBDGs. However, by offering low‐threshold, individualized interaction without requiring formal enrolment or human interaction, the tool could contribute to reducing the gap between abstract dietary recommendations and their practical implementation.

This study provides qualitative evidence that the German FBDGs are broadly comprehensible but fall short in supporting real‐life dietary implementation. The disconnection between nutritional knowledge and behavioral application—observed across participants regardless of their existing knowledge or motivation—suggests that informational approaches alone are insufficient to bridge this gap. The conceptual AI‐based feedback tool presented in this study was received positively and represents a promising direction for more practical, individualized nutrition communication. However, its potential to induce meaningful behavior change remains to be tested empirically.

More broadly, this study raises a question of responsibility: if national organizations such as the DGE rely primarily on FBDGs as their instrument of nutrition promotion, and if such informational tools appear insufficient to translate into behavioral change, then the development of complementary, behavior‐oriented programs appears not merely desirable but necessary. Future research should prioritize the empirical testing of digital tools like the one presented here and explore how such approaches can be integrated into national nutrition strategies to address the practical realities of everyday eating.

## Author Contributions

**Philipp Sprengholz:** conceptualization, methodology, writing – review and editing. **Nicolas Hartmann:** conceptualization, methodology, formal analysis, investigation, writing – original draft.

## Funding

This work was supported by the University of Bamberg.

## Conflicts of Interest

The authors declare no conflicts of interest.

## Data Availability

Original German versions of the quotes presented in this article are available at https://doi.org/10.17605/OSF.IO/G6P7S. Full interview transcripts will be made available by the authors upon request.
